# Torturing and Starving Fish

**Published:** 1889-08-24

**Authors:** J. Laurence-Hamilton


### Torturing and Starving Fish.

By J. Lawrence-Hamilton, M.R.C.S.

It is sad to relate that in the fish trades of the United Kingdom those professionally and pecuniarily interested and engaged in fish torturing and in fish starving have, in the ignorant thoughtlessness of chronic custom, been alike blinded to the sacred civilising duties of humanity and Christianity, and at the same time have overlooked and omitted their own material profits and advantages. I shall show that fish starving is a domestic sport which simultaneously diminishes the commercial value of the fish; it ruins the flesh, flavour, and firmness of the fish; it tends to induce parasitic, bacterial, and other diseases in the fish; it diminishes the weight of the fish, and increases its cost both to the professional fish starver and to the consumer—the general public. Fish so starved are more liable to rapid decomposition soon after death, and in codfish such starved fish are more difficult to crimp, which are additional causes and circumstances tending to enhance the avoidable risks of the present fish trade, and therefore an unfair tax on the consumer. The average absolute loss in weight of codfish by starvation and imprisonment appears to represent 10 per cent. of the entire weight of the natural normal healthy fish, a heavy avoidable commercial loss. The close confinement of codfish in their fish-chests sunk in the fish docks causes a rapid increase in the relative number of bacteria always present in the blood of living fish, and when the proportion of bacteria exceeds that which the blood of living fish can tolerate, then the fish commences to sicken and will often die, even if well and regularly fed in a fair amount of space, as in the well-cared-for fish kept in aquaria but too often illustrate. Hence it is that these sunk fish-trunks become the rapid carriers of contagious disease to any healthy fish which may chance to be in or near the fish-chest.

It is known that fish, in addition to tolerating in health a certain proportion of bacteria, are also, up to a certain point, equally tolerant of various external and internal parasites, and generally every fish dissected shows either some parasites—as tapeworms—in its intestines, or other parasites about its gills, eyes, skin, or elsewhere, and even sometimes as many as fifteen different species of parasites have been discovered in one individual fish. Often these parasites, along with the bacteria, increase so as to gain the battle over the fish, especially when the latter becomes weakened by starvation, confinement, injury, or other causes tending to diminish its vitality. Parasites also promote decomposition the moment the fish dies. These sunk fish-chests never err on the side of cleanliness, and are hence favoured localities for hatching and breeding bacteria and parasites specially destructive to fish health and fish life. Later I hope to be able to publish a detailed illustrated pamphlet on this subject. The loss of life in codfish caught in the nets will not average upwards of 5 per cent. Hooked fish on being drawn up are liable to disgorge or vomit not alone the contents of their stomachs, but also, especially in the case of deep-sea and abyssal fishes, to have their stomach and part of their intestines protruded through their mouths. Deep-sea and abyssal fishes, fish having air bladders, by the rapid altered pressure, together with the influence of shock, get their air bladders so dilated with evolved gases that they are unable to sink from the surface of the water in welled smacks till the fisherman, passing a long needle through the walls of the fish's abdomen, or the pit of the left pectoral fin, by letting the gases escape, allows the fish to resume its natural habits. Hooked salmon vomit through fright alone. Fish may be sometimes seen with a prolapsed and protruded rectum and lower intestine, which appears to be due to relaxed flabby muscles, and is a sign which very shortly precedes the commencement of decomposition. As a rule, hooked fish keep longer fresh than netted fish. Netted fish, owing to being long injured by having been trawled over rough grounds, together with the sharp prolonged squeezing and banging about which they get in the nets, are, like over-driven cattle, specially liable to early decomposition.

Much of the so-called best prime "live" codfish at Billingsgate has after capture been transferred into welled smacks, which accommodate from about 600 to 1,000 codfish, each weighing from 16lb. to 20lb. avoirdupois, where, especially in rough weather, after having been subjected for a few days to injury, confinement, and starvation in the welled boats, to prevent these fish from eating one another they are sometimes specially secured by their tails. On arriving in port some forty of these codfish are tightly crammed into a fish-chest, which is sunk in the fish dock (which in England answers only during the cold weather, from about November till April), where the codfish, after further confinement and starvation, all die within a few weeks. If the fish be kept for a shorter interval—say, a fortnight—then on an average only about 10 per cent. of such codfish are found dead upon opening the raised chest. All such starved, closely-confined codfish lose much of their natural healthy flavour, firmness, weight, and plumpness. The fish having to support life, consume much of the substance of their own livers, which appear small and shrunk when compared with the normal liver of a freshly-killed healthy fish. Consideration of the chemical and microscopic changes thus artificially induced, I propose to postpone for a subsequent occasion. This codfish, if not previously dead, is then stunned with a blow from a big mallet, and thus, whilst insensible, crimping is alleged to be carried on. As the sensibility never returns there is practically no cruelty. Skate are said sometimes whilst still alive to be crimped by fishmongers. So long as cod will "crimp," or show muscular contraction, it is called in the fish trade "live cod," an intentional misrepresentation. Out of its healthy season, a woolly, watery codfish is often appropriately termed by the fishermen "churchyard" cod. Recollecting these facts, the familiar dry, flabby, cotton, wool-like taste of the vaunted best prime "live" codfish so often sold at Billingsgate and elsewhere is easily explained. Skinning live eels and gutting live fish, causing avoidable, useless torture, are occasionally practised.

Lobsters and crabs are often kept alive on wet seaweed or ferns, and live on an average in hampers for a couple of days, starving before they reach the fishmonger, who frequently keeps them, if not sold, till they die of starvation. Lobsters and crabs thus starved to death lose weight, firmness, taste, and flavour, and immediately on death tend to decompose rapidly, which in such conditions is but slightly diminished by boiling in salted water. Instead of puncturing their brains with a stilleto, wriggled about so as to break up the animal's brain material, and then plunging the crabs and lobsters into boiling water, out of sheer thoughtlessness and careless custom, living lobsters have been placed in cold water which, whilst it is being heated up to the boiling point, causes the animal unnecessary and easily avoidable torture, for if plunged into boiling water it quickly dies. However, previously to boiling crabs and lobsters, to rid them of sand and dirt they should be well washed in fresh flowing water. Although no fish or shell-fish is legally protected against cruelty in this country, and although it is not punishable to boil fish alive, to roast fish alive, to slice fish alive (as turtle used to be in Ceylon, and as some air-breathing fish carried about in earthen pots in China are still), yet it is to be expected that these cruelties will rapidly disappear as soon as fish traders find that humanity to animals will benefit their own pockets. Codfish would keep in excellent health and condition if kept free in artificial ponds or docks fed with sea water, as was the custom among the ancient Romans and ancient Egyptians. The present St. Petersburg fish farms, or ponds, fed by the River Neva, and where each species of fish has a separate compartment, yield profitable rentals. Lobsters and crabs can be fed on almost any fish refuse. The English law is curious as regards cruelty to animals. Scratching a tame rabbit so as to draw its blood is punishable with three months' imprisonment with hard labour, whilst the law permits anyone to roast a *wild* rabbit alive without any interference. Possibly the Legislature considers fish as wild animals, and therefore beyond and outside the jurisdiction of the law courts.—*The Lancet.*

